# Experimental infection of ringtail possums (*Pseudocheirus peregrinus*) with *Mycobacterium ulcerans*, the agent of Buruli ulcer

**DOI:** 10.1038/s41598-024-76857-1

**Published:** 2024-10-25

**Authors:** Kim R. Blasdell, Richard J. Ploeg, Emma C. Hobbs, Stephen Muhi, Sarah J. Riddell, Alexandra Cunneen, Michael L. Kelly, Kate Maynard, Tess R. Malcolm, Md. Tanjir Islam, Victoria Boyd, Timothy P. Stinear, Sacha J. Pidot, Eugene Athan, Daniel P. O’Brien

**Affiliations:** 1 Health and Biosecurity, Commonwealth Scientific and Industrial Research Organisation, Geelong, Australia; 2 Australian Animal Health Laboratory, Commonwealth Scientific and Industrial Research Organisation, Geelong, Australia; 3 Department of Veterinary Biosciences, University of Melbourne, Melbourne, Australia; 4 Peter Doherty Institute for Infection and Immunity, University of Melbourne, Melbourne, Australia; 5 Department of Infectious Diseases, Barwon Health, Geelong, VIC Australia

**Keywords:** Skin diseases, Bacterial pathogenesis, Infectious-disease epidemiology, Pathogens

## Abstract

Buruli ulcer (BU) is a necrotizing disease of skin and soft tissue caused by the bacterium *Mycobacterium ulcerans* (MU). In Australia, where the disease is emerging in new geographic areas and human case numbers are increasing, native possum species act as reservoir hosts. To better understand the life history of MU in one of its natural hosts, we conducted intra-dermal challenge of six wild caught, MU-naïve common ringtail possums (*Pseudocheirus peregrinus*). All six animals developed BU disease consistent with that observed in naturally infected ringtail possums. Time to ulceration varied between 49 and 77 days (mean = 61.8 days). Molecular evidence of systemic infection was detected in five animals and was supported by consistent histopathological findings in four animals. Pathological findings included random, multifocal, granulomatous hepatitis in four possums, one of which also had a mild, multifocal, interstitial granulomatous pneumonia. Acid-fast bacilli were only evident in inflammatory foci beyond the primary inoculation site in one possum. The ringtail possum model of MU infection is an important tool for the investigation of bacterial transmission dynamics, pathogenesis and immune response in a natural host. Data from this model may improve disease risk modelling and help identify intervention points to stop zoonotic transmission and disease spread.

**Supplementary Information:**

The online version contains supplementary material available at 10.1038/s41598-024-76857-1.

## Introduction

*Mycobacterium ulcerans *(MU) is the agent of Buruli ulcer (BU), a necrotizing disease of subcutaneous tissues. Infection is thought to occur through skin micro-trauma and often initially manifests as a small nodule, which can progress to a large necrotic ulcer if left untreated, leading to potential deformity and significant morbidity^1,2^. Although BU disease in humans is treated through surgery and/or combination antibiotic regimens, the former is expensive, and the latter is associated with paradoxical reactions in a substantial proportion of patients^3^. Alongside the physical aspects of this disease, BU has emotional and psychological impacts on patients and their carers, and the risk of infection can generate fear in endemic communities^4^. This predominantly tropical disease is found worldwide, with most cases reported in western Africa and southern Australia^5^. Although generally considered a rural disease in Africa, in Australia, most cases occur in urban and suburban areas in the temperate southern state of Victoria^6^. In Victoria, where this disease is notifiable in humans, human case numbers have been increasing and the endemic area expanding, with local transmission recently identified in parts of the state’s capital, Melbourne, and its second-largest city, Geelong^7,8^.

In Africa, evidence for the involvement of non-human mammals in the environmental circulation of MU is limited^9^. In contrast, in Victoria at least two species of native marsupial, the common ringtail possum (*Pseudocheirus peregrinus*) and common brushtail possum (*Trichosurus vulpecula*), are commonly infected and are therefore considered reservoir hosts of the bacteria^10,11^. Both possum species shed MU in their feces and have been found with MU positive cutaneous lesions, with evidence of systemic disease identified in some individuals^12,13^. Although direct transmission of MU from possums to humans appears to be rare, at least one case of bite-based transmission has occurred^14^.

MU DNA has been detected in numerous environmental sample types, but only feces from ringtail and brushtail possums and red foxes (*Vulpes vulpes*) have been found to contain potentially viable bacteria^11^. The distribution of MU DNA-positive possum feces is positively associated with the distribution of human BU cases, and potentially predictive of future human BU cases^7^. Environmental characteristics conducive to possum presence (e.g. overhead powerlines, native tree species) are also associated with human cases^11^. How and if transmission is occurring between possums and humans is still unknown but can involve a mosquito vector, although introduction of organisms into the skin by other forms of puncturing injury such as bites or scratches may also be possible^1,14–16^. Comparisons of MU whole genome sequences from humans and possums have shown they are genetically indistinguishable^10^.

Several animal models of disease already exist for MU, including mouse, guinea pig and pig models^17^, although none are natural hosts of BU. A variety of other species have also been successfully challenged with this pathogen including brushtail possums^18^. However, a model of disease under controlled conditions in a natural host species has yet to be attempted. The development of such a model would aid investigation of potential transmission routes, informing ecological and epidemiological risk analyses of disease distribution and spread, as well as pathogenesis and immune responses. It would also provide opportunities for trialing possum-based BU intervention measures, such as oral-bait vaccination^19^. Here we present the results from the first experimental challenge of common ringtail possums with MU and demonstrate that replicable disease can be produced in this species.

## Results

### Trapping and acclimatization of wild ringtail possums

A total of eight ringtail possums were captured over a one-week period. All animals were visibly healthy as assessed by a veterinary professional. Two animals (both adult females) tested positive for MU DNA during initial screening testing, but as there are no established treatment or management protocols for BU in possums and no clinical signs were present, euthanasia was not indicated and both animals were later released at their respective capture sites. The remaining six animals (two adult females, four adult males) were negative and were transferred to the Large Animal Facility (LAF) at the Australian Centre for Disease Preparedness. Animals were acclimatized for between three to four weeks. All animals adapted well to the captive environment as assessed by overnight video monitoring, food consumption, and fecal production.

### Buruli ulcer disease development in experimentally *M. ulcerans*-challenged ringtail possums

**Fig. 1:**
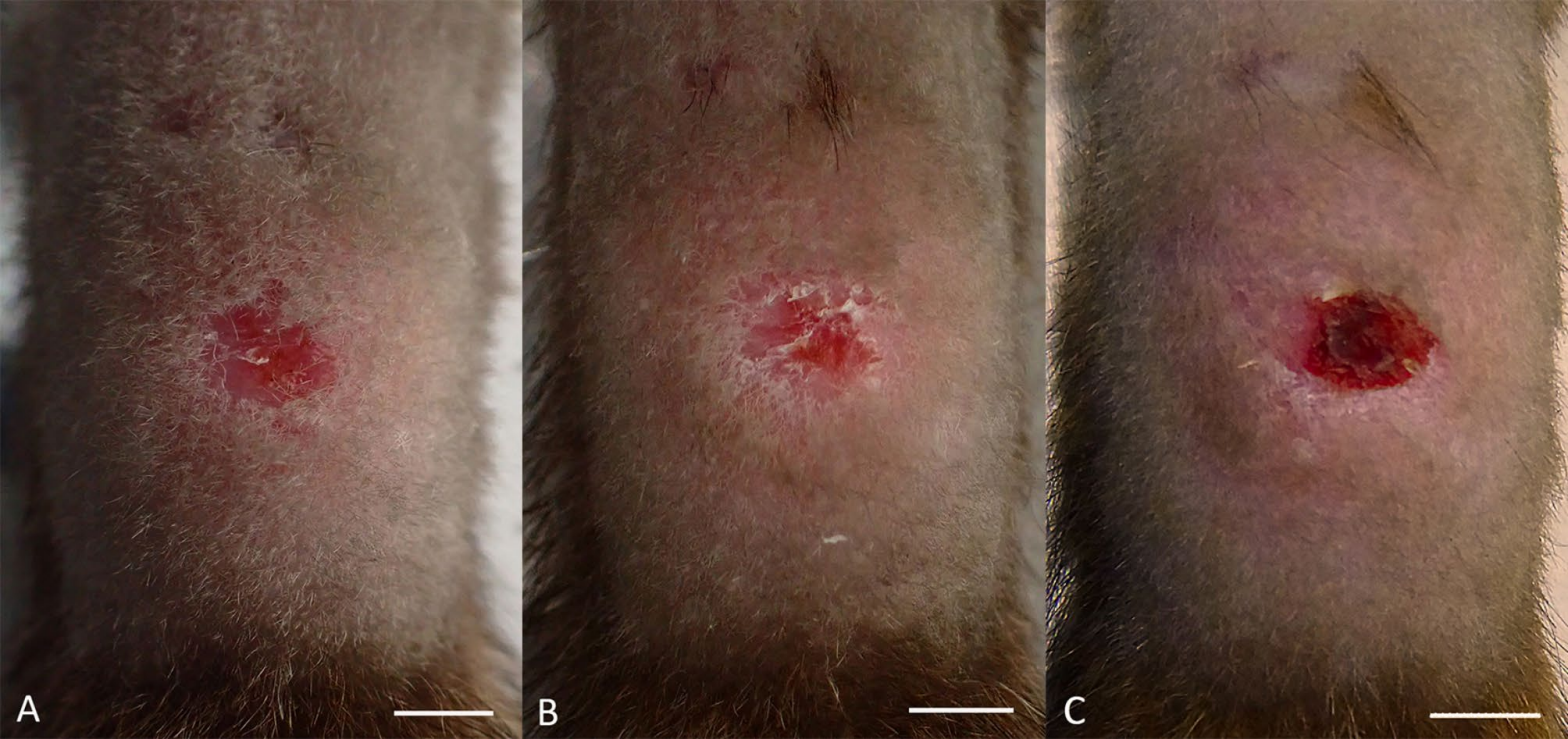
Gross appearance of lesions at MU inoculation site. (**A**) Possum #5 60 DPI. Pre-ulcerative lesion demonstrating regional swelling, hair loss and hyperaemia. Scale = 5 mm. (**B**) Possum #5 70 DPI. Pre-ulcerative lesion demonstrating regional swelling, hair loss, hyperaemia, scaling and superficial erosion of the skin. Scale = 5 mm. (**C**) Possum #5 74 DPI. Ulcerative lesion demonstrating regional swelling, hair loss, and ulceration with eschar formation. Scale = 5 mm.

All six animals developed clinical signs consistent with MU infection. All initially developed a swelling at the site of challenge, i.e., on the dorsal mid-tail region: four on 39 days post-infection (dpi), one on 53 dpi and one on 60 dpi (mean = 45 dpi, SD 9.3 days) (Table 1). Overlying these pre-ulcerative swellings were well-defined areas of hair loss, thinning of the skin and superficial erosion (Fig. 1). The swelling at the challenge site went on to ulcerate in four of the six animals (Fig. 1), whilst additional pre-ulcerative lesions or ulcers developed distal to the challenge site in the remaining two animals (Table 2). Animal #1 was the only animal to develop more than one ulcerated lesion, with two small ulcers developing on the tail distal to the challenge site. Most ulcers were approximately round in shape, although animals #4 and #6 developed an irregular- and an oblong-shaped ulcer respectively. Ulcers varied in size from 1 mm x 1 mm to 16 mm x 7 mm (Table 3).

**Table 1:** Characteristics of BU disease in experimentally *M. ulcerans*-challenged ringtail possums, by animal.

Animal #	Sex	DPI clinical onset	DPI ulceration	DPI euthanasia	Time between clinical onset and euthanasia (days)	Reason for euthanasia	DPI oral swabs positive	DPI feces positive	Systemic infection
1	Female	39	60	60	21	Ulceration	5, 12	None	No
2	Female	60	N/A	67	7	Drop in body condition	None	None	Yes
3	Male	39	77	77	38	Ulceration & drop in body condition	5	67, 74, 77	Yes
4	Male	39	49	49	10	Ulceration	None	None	Yes
5	Male	53	74	74	21	Ulceration	None	None	Yes
6	Male	39	49	49	10	Ulceration	5	None	Yes
Mean	.	44.8	61.8	62.7	17.8	.	.	.	.
SD	.	9.3	13.3	12.1	11.5	.	.	.	.
Variance	.	86.6	177.7	146.7	133.4	.	.	.	.

*DPI* days post infection,* SD* standard deviation.

**Table 2:** Characteristics of ulcers and pre-ulcerative lesions, including final dimensions at euthanasia, in experimentally *M. ulcerans*-challenged possums, by animal.

Animal #	Sex	Ulcer or lesion	Ulcer/lesion dimensions		Ulcer/lesion shape	Ulcer/lesion location
			Length (mm)	Width (mm)		
1	F	Ulcer (x2)	1 (both)	1 (both)	Round (both)	Both distal to challenge site
2	F	Pre-ulcerative lesion	4	4	Round	Distal to challenge site
3	M	Ulcer	3	3	Round	Challenge site
4	M	Ulcer	5	4	Irregular	Challenge site
5	M	Ulcer	4	5	Round	Challenge site
6	M	Ulcer	16	7	Oblong	Challenge site

**Table 3:** Evidence of systemic MU infection in experimentally challenged ringtail possums.

	Tissues positive for MU by IS2404 PCR, with (C_T_ values)						
Animal #	Ulcer	Heart	Blood	Lung	Liver	Gall bladder	Evidence of systemic MU infection
1	Y (33.7, 29.2, 35.3)^^^	N	N	N	N	nt	No
2	Y (25.7)	Y (38.1)	Y (35.5)	Y (37.0)	Y (38.5)	nt	Yes (molecular & pathology)
3	Y (29.2)	N	N	N	N	nt	Yes (molecular & pathology)
4	Yn (27.2)	N	N	N	N	nt	Yes (molecular & pathology)
5	Y (28.5)	N	N	Y* (39.6)	N	Y (38.1)	Yes (molecular & pathology)
6	Y (27.0)	Y (34.9)	N	Y (39.6)	N	nt	Yes (molecular only)

	Tissues positive for MU by IS2404 PCR, with (C_T_ values)						
Animal #	Stomach	Large intestine	Small intestine	Caecum	Kidney	Inguinal lymph node	Evidence of systemic MU infection
1	N	N	N	N	N	nt	No
2	N	Y (35.1)	N	N	N	nt	Yes (molecular & pathology)
3	N	Y (37.5)	Y (39.6)	N	N	Y (39.8)	Yes (molecular & pathology)
4	N	Y (37.6)	Y (38.6)	N	N	Y (36.9)	Yes (molecular & pathology)
5	N	Y (39.9)	N	N	N	Y (39.7)	Yes (molecular & pathology)
6	Y (39.9)	N	N	N	N	nt	Yes (molecular only)

*nt* not tested; *FFPE sample used for histology. ^^^Three lesions were present in this animal with C_T_ values listed in the order: challenge site pre-ulcerative lesion, mid-tail distal to challenge site ulcer, tail tip ulcer).

Although body weight remained relatively stable for all animals throughout the study, two animals (#2 and #3) experienced a drop in body condition leading to euthanasia (Table 1; Supplemental Fig. 1). Both inter- and intra-animal daily fecal production was highly variable throughout the study. However, four animals (#1, 4, 5 and 6) each appeared to experience a temporary drop in fecal production for several days post-challenge but prior to the development of clinical signs (Supplemental Fig. 2). No obvious trends were observed for either rectal or microchip temperatures (Supplemental Fig. 3).

Animals were euthanized between 49- and 77-dpi due to ulceration and/or a drop in body condition, with the duration between clinical onset and euthanasia being considerably variable (10 to 38 days, mean = 17.8 days) (Table 1). Animal #3 survived the longest after the onset of lesion development (38 days) and was the only animal to show evidence of MU fecal shedding, from day 67 dpi. Fecal pellets from all other animals remained PCR negative throughout the study. MU DNA was detected in the oral swabs of three animals (#1, 3 and 6) for one to two weeks post-challenge and then became negative, which may have reflected the animal’s grooming of the challenge site (Table 1).

### Pathology and systemic infection

**Fig. 2:**
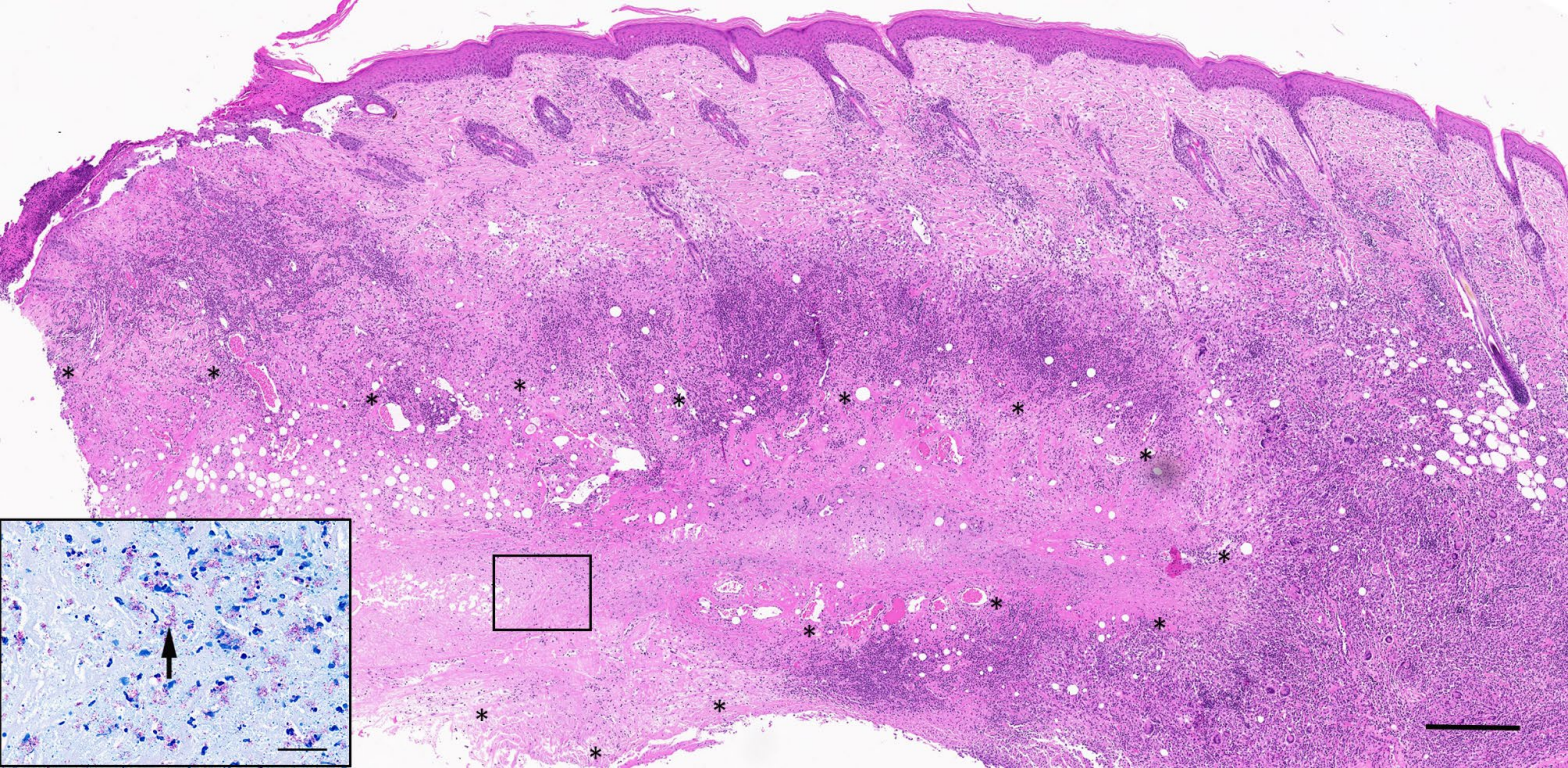
Histology of MU inoculation site. Main: Possum #5, 74 DPI. Locally extensive region of deep dermal and subcutaneous necrosis roughly outlined with ‘⁎’) with a surrounding mantle of mixed inflammatory cells including frequent multinucleated giant cells. Inflammation extends into the overlying dermis and there is intracorneal pustule formation and sub-epidermal clefting of the overlying epidermis. Haematoxylin and eosin. Scale = 500 μm. Inset: Numerous, largely extracellular, magenta-staining, AFB. Wade-Fite modification of Ziehl-Neelsen stain. Scale 50 μm.

**Fig. 3:**
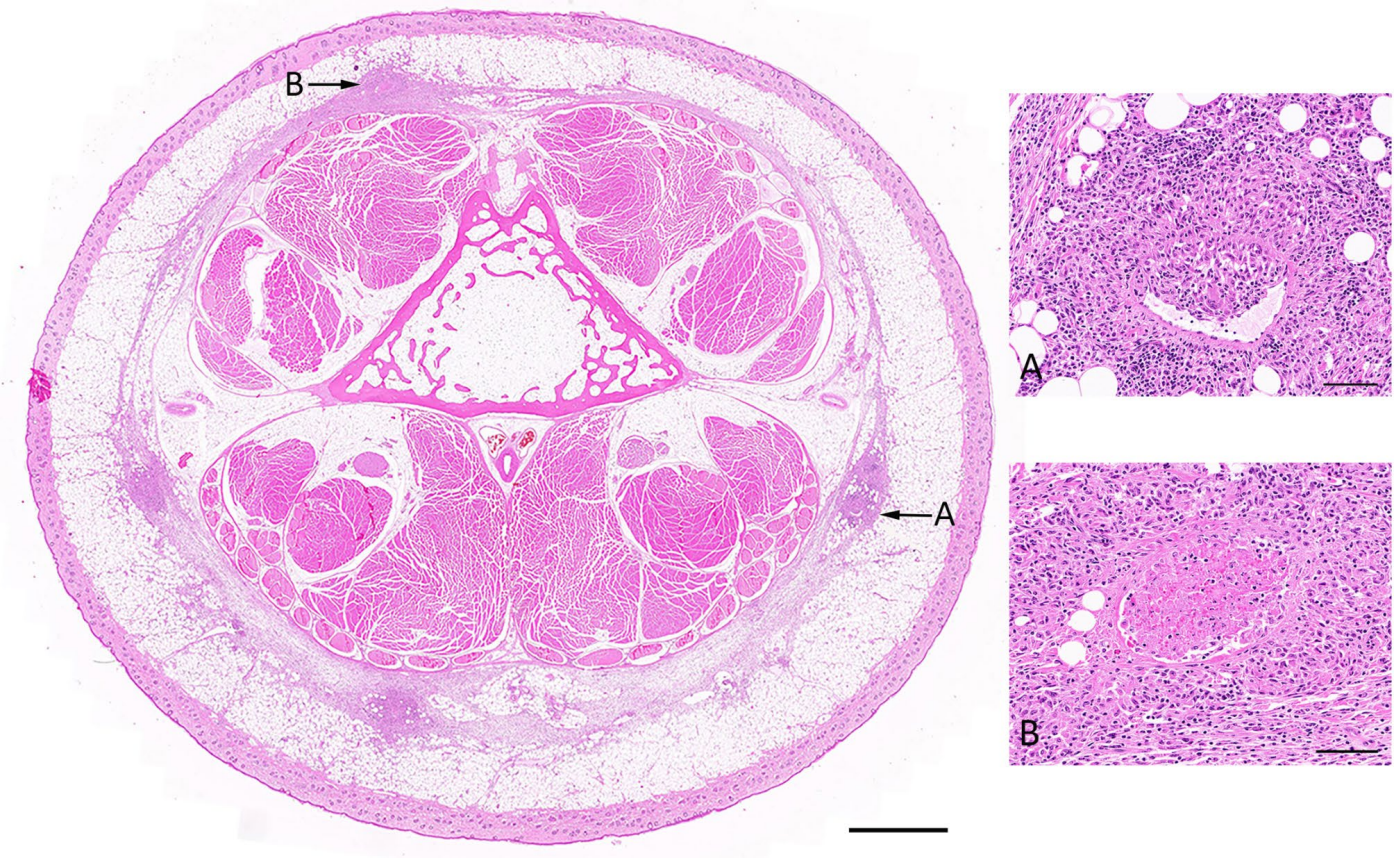
Histology of a cross-section of the tail base several centimetres proximal to the primary MU inoculation site. Main: Possum #2, 67 DPI. A cross section through the proximal tail revealing multiple foci of subcutaneous inflammation, two of which are highlighted with arrows. Haematoxylin and eosin. Scale = 2 mm. Inset (**A**) Section through an inflamed lymphatic vessel revealing a dense mantle of encircling macrophages (including multinucleated giant cells) and lymphocytes. Haematoxylin and eosin. Scale = 100 μm. Inset (**B**) Section through an inflamed and presumably thrombosed blood vessel (venule) bearing accumulations of necrotic cellular debris intermixed with erythrocytes and encircled by a mantle of macrophages and lymphocytes. Haematoxylin and eosin. Scale = 100 μm.

A necrotizing, pyogranulomatous to granulomatous dermatitis and panniculitis was evident at the site of MU inoculation in all six animals (Fig. 2). This was represented by locally extensive areas of necrosis surrounded by a variable mantle of neutrophils and macrophages, including multinucleated giant cells (Langhans and foreign body type), as well as numerous lymphocytes and lesser numbers of plasma cells. Inflammation typically extended to involve the tendon sheaths, and, at times, skeletal muscle underlying the inoculation sites, and also proximally along the tail within the subcutaneous adipose tissue, lymphatic vessels (lymphangitis) and in one animal (#2), a blood vessel. Most often evident at the margins of the regions of necrosis in each of the animals were scattered small to medium-sized blood vessels with walls bearing frequent neutrophils (vasculitis) and at times effaced by accumulations of amorphous eosinophilic material (fibrinoid necrosis) with intra-luminal fibrin thrombi. In all animals varying degrees of epidermal erosion and occasionally intraepidermal pustule formation were noted. Whilst the epidermis overlying the primary necrotizing lesion was typically acanthotic and hyperplastic, in four animals (#3, 4, 5, and 6) and evident in Fig. 2, there was also evidence of overlying follicular atrophy and loss as well as patchy clefting at the dermo-epidermal junction, implying some degree of regional ischemia. Although a granulomatous lymphangitis at the primary inoculation site was seen in all cases, it was also noted in four animals (#1, 2, 5 and 6) at the proximal margin (root) of the tail several centimeters from the inoculation site (Fig. 3). This cross section of the tail from possum #2 revealed numerous foci of granulomatous inflammation centered on subcutaneous lymphatic vessels (Fig. 3, Inset A). Also noted in the tail cross section from possum # 2 was a distended and presumably thrombosed blood vessel bearing necrotic cell debris intermixed with erythrocytes and surrounded by a significant infiltrate of macrophages and lymphocytes (Fig. 3, Inset B). This vasocentric lesion was the only inflammatory focus that incorporated a region of necrosis beyond the primary inoculation sites.

**Fig. 4:**
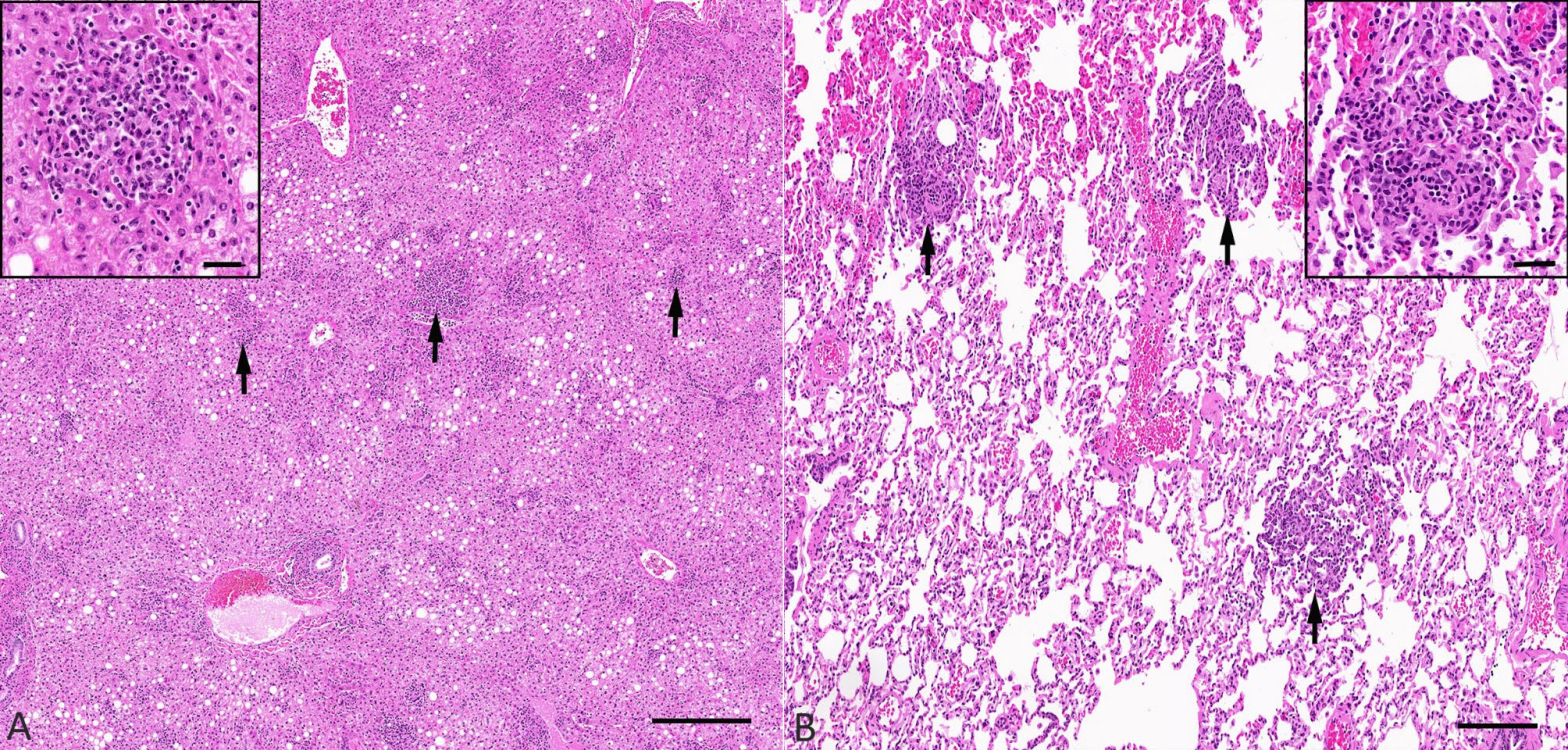
Histology of hepatic (**A**) and pulmonary (**B**) lesions. (**A**) Main: Possum #5, 74 DPI. Randomly and multifocally throughout the liver were variably-sized inflammatory foci (arrows) as well as a mild to moderate periportal infiltrate of similar composition. Haematoxylin and eosin. Scale = 500 μm. Inset: An intra-sinusoidal accumulation of macrophages and lymphocytes as highlighted by the arrows in the main image. Haematoxylin and eosin. Scale = 50 μm. (**B**) Possum #5, 74 DPI. Multifocal expansion of the pulmonary interstitium by focal accumulations of macrophages and lymphocytes (arrows). Haematoxylin and eosin. Scale = 250 μm. Inset: Focal expansion of the pulmonary interstitium by accumulation of macrophages and lymphocytes as highlighted by the arrows in the main image. Haematoxylin and eosin. Scale = 50 μm.

In three animals (#2, 3 and 5) there was moderate, multifocal hepatitis as shown in Fig. 3. This was characterized by random, variably- sized, intra-sinusoidal as well as frequent periportal accumulations of inflammatory cells (Fig. 4) represented by macrophages with intermixed small lymphocytes (Fig. 4). In one animal (#5) there were also scattered, multifocal, interstitial accumulations of macrophages and lymphocytes in lung tissue (Fig. 4). No other gross pathological findings were evident in any animals.

As highlighted in the inset of Fig. 2, numerous extra-cellular and rare intra-cellular acid-fast bacilli were evident within pre-ulcerative and ulcerated lesions at the MU inoculation sites, concentrated in the regions of necrosis. In one possum (#2), rare acid fast bacilli (AFB) were evident amidst an intraluminal accumulation of necrotic cell debris in a vasocentric granulomatous inflammatory focus at the base of the tail (Supplemental Fig. 4). As previously mentioned, this lesion, along with the adjacent foci of lymphangitis, was several centimeters proximal to the point of MU delivery. Other than this vascular lesion, there was no evidence of intralesional necrosis in foci of inflammation distant to the primary lesion.

Molecular evidence of systemic infection was confirmed in all animals except #1, as determined by the detection of at least one internal tissue positive by IS*2404* PCR (Table 3). These findings were supported by histopathological analysis of tissue samples collected from four of the six possums, with evidence of a random, multifocal, granulomatous hepatitis in possums #2, 3, 4, and 5 and a mild, multifocal, interstitial granulomatous pneumonia in possum #5. In addition, possum #2 was the only animal in which the blood was PCR positive for MU and histology confirmed vascular invasion in this case (Fig. 3, Supplemental Fig. 4).

### Immunological detection of possum seroconversion to *M. ulcerans*

With evidence of MU-consistent infection in all possums, we sought to investigate seroconversion of the animals through immunological blotting methods. An initial SDS-PAGE analysis of MU whole cell lysate showed several well-separated proteins spanning between approximately 15 to 150 kDa (Supp. Fig. XA). No interaction between possum serum and SDS-PAGE-separated MU proteins was detected using Western Blotting. Using the Dot Blot method a response to MU was detected at both 1:10 and 1:100 dilutions of the necropsy serum from possum #3. Signal response increased between the 1:100 and 1:10 dilutions, showing that an increase in serum material results in higher signal and suggesting a true interaction between serum and MU (Supplemental Fig. 5). However, a signal comparable to the 1:100 necropsy serum sample was also observed from the no serum control and Possum #3 pre-challenge serum (Supplemental Fig. 5). Whether this response signal occured due to an interaction between pre-infection serum and MU, or cross-reactivity between Protein A and MU was not pursued as part of this study.

## Discussion

Common ringtail possums have been implicated as reservoir hosts of MU in Victorian endemic areas and likely contribute to both the bacteria’s ecology and the epidemiology of BU in this region^10,11^. Although evidence of infection, including possible systemic disease, has been identified in wild possums previously^12^, this study represents the first attempt to develop a ringtail possum model of MU infection. Intradermal challenge of captive-housed wild ringtail possums led to disease in all animals that was comparable to that observed in naturally infected possums, and enabled characterization of early-stage BU disease progression in this species.

Disease development in our experimental ringtail possums was similar to the progression observed in humans and other animal models of BU. Although a slight increase in body temperature was recorded in some animals around the onset of clinical signs, values remained within the normal range for this species^20^. Fever has not been recognized as a common feature of BU disease in either humans or other animals^21^. Body weight also remained relatively stable throughout the experiment, although two animals did experience a drop in body condition just prior to euthanasia, which may have been an early indication of the considerable morbidity and mortality that ringtail possums experience under natural conditions^13^. A recent study of four severely diseased ringtail possums naturally infected with MU found that all animals had a poor body condition score (2 out of 5 or lower)^13^. Weight loss has also been observed previously in a horse naturally infected with MU and in human patients with unusual BU disease manifestations^22–24^, but not in most human patients or in experimentally infected rats or mice^25,26^.

Infected humans do not shed MU DNA in their feces^27^ and whilst fecal shedding has been observed in rats, mice and naturally infected possums, the impact of MU infection on fecal production has not been assessed previously. As these wild animals were captive-housed and fed a diet that was not entirely reflective of what they would consume under natural conditions, it is possible that this may have caused the drop in fecal production seen in four of the animals. However, all animals had already been captive-housed for several weeks during the acclimatization phase, with no similar reduction in fecal output observed prior to MU challenge. Instead, the reduced fecal output occurred approximately two weeks post-challenge in three of the four animals, and in the fourth animal, at three weeks post-challenge and so may have been related to the onset of systemic disease. Further study would be needed to investigate whether this is indeed a result of MU infection.

Unfortunately, the presence of MU-specific antibodies could not be detected using Western Blot or Dot Blot methods, likely due to incompatibility with the secondary antibody used (Protein A). An article published after the completion of the current study demonstrated that Protein A/G (of which Protein A is a component) does not bind to ringtail possum antibodies^28^. Interestingly, although all six animals in the current study developed swelling at the site of challenge, the ulcers or pre-ulcerative lesions that later developed did so at the site of challenge in all male animals (*n* = 4) but were distal to the challenge site along the tail in both female animals (*n*= 2). It was in one of these animals (#2) that AFBs were detected at the tail base, proximal to the inoculation site, suggesting localized MU spread, apparently via the hematogenous route. However, due to the small sample size used here, further investigation would be needed to establish if biological sex truly has an impact on lesion site development in ringtail possums. A study of naturally infected animals did find that male ringtail possums were more likely to have clinical lesions than females, but this could be related to sex-related behavioral differences, rather than physiological differences^12^.

The time to the onset of lesion development (mean = 6.4 weeks) and cutaneous ulceration (mean = 8.8 weeks) reported here was comparable to that observed in other animal models, despite the relatively low challenge dose used in this study (506 CFU). BALB/c mice challenged with a lower dose (9–55 CFU) of the same Australian MU strain used in this study (JKD8049) developed lesions between 6 and 24 weeks post-challenge and had an estimated median incubation period of 12 weeks^15^. In another study using a bioluminescent-expressing variant of the same MU strain, BALB/c mice subcutaneously challenged with a much higher dose (3.3 × 10^5^CFU) reached humane endpoint between 8- and 17-weeks post-challenge^29^. Mice challenged with high doses of African MU strains have been found to have slightly faster disease development, with swelling observed by 4 weeks and ulceration by 7 weeks post-challenge^30,31^. In the pig model, subcutaneous challenge with a high dose of a MU strain from Cameroon (2 × 10^7^CFU) resulted in macroscopic changes in the skin by 2.5 weeks and induration or ulceration by 6.5 weeks^32^. In a study conducted in Australia in the 1950s, several common brushtail possums were inoculated either subcutaneously in the thigh or intra-peritoneally with an unknown dose of MU^18^. Although time to clinical onset was not reported for all animals, where this was recorded, ulceration appeared to occur approximately four months post-challenge. Spontaneous healing of lesions was observed in some of these animals, as also seen in some wild brushtails^12^. However, as these animals were lost to long-term follow up it is unclear whether infection was completely cleared or whether animals undergo cycles of ulceration and resolution.

Evidence of MU DNA was detected in the pre-ulcerative and ulcerated lesions of all our study animals, as has also been reported in naturally infected possums^12,13^. Molecular analysis also detected systemic distribution of MU DNA in five of our study animals: at least one region of the gastrointestinal tract tested positive by real time PCR in all five animals; inguinal lymph nodes were positive in the three animals for which these were tested; lung was positive in three animals; heart and liver/gall bladder were each positive in two animals; and blood in one animal. Evidence of gastrointestinal tract involvement has been identified previously in naturally infected wild possums, with one study detecting MU DNA in either the gut contents or stomach wall of 7 out of 27 infected animals^12^. Other organs also appeared to be affected in four of these wild ringtail possums including liver and lung in four animals; kidney and spleen in three animals; and heart, mandibular lymph node and mesenteric lymph node in one animal. In another study of four severely diseased possums, MU DNA was detected in all organs tested suggesting widespread systemic infection^13^. In contrast to naturally infected animals, no evidence of kidney involvement was detected during the current study. The inflammatory patterns observed in liver (possums #2, 3 and 5) and lung (possum #5) sections would suggest hematogenous spread of MU occurred in these animals (no liver was available for histology for possum #4). Hematogenous and/or lymphatic dissemination of MU has also been reported in experimentally infected mice and grasscutters (*Thryonomys swinderianus*)^17,33^, although more research is needed to further investigate the local and systemic effects of MU in infected hosts.

No AFB were observed on stained lung or liver sections from any of our study animals, which is also typical for tissues of naturally infected possums^12,13^, although rare AFBs were observed within macrophages from liver samples of four wild infected ringtails^12^. It has not been established whether the absence of observable MU organisms in the PCR-positive tissues of infected animals reflects a true absence of the bacteria in these organs, an absence of viable bacteria, or rather an inadvertent sampling bias. Of interest in this study was the detection of AFB within the lumen of a blood vessel distant to the primary inoculation site and that these intact organisms were associated with the only focus of cellular necrosis away from the point of MU delivery. As such, in this study, intact AFB were only detected in inflammatory foci incorporating significant intralesional necrosis and would seem likely to reflect the presence of enough intact organisms to manifest this change via exotoxin production. The presence of intact AFB within a blood vessel is also of interest in terms of potential transmission pathways. This could provide a source of contamination (or infection) of mosquitoes during a blood feed, lending further support to the hypothesis that MU is a mosquito-borne pathogen^16^.

In the current study, all organ samples positive for MU DNA had C_T_values of approximately 35 or higher, indicating that if intact AFBs were present, they were at low frequency and may have been missed by histology. In addition, animals were euthanized at the point of ulceration, which may have limited the amount of systemic MU dissemination. It is suspected that MU disease is progressive in ringtail possums. Under natural conditions, infected possums likely survive for several months after ulceration, which would provide greater opportunities for bacterial dissemination, colonization of additional organs, and disease progression as observed in several severely diseased animals^13^. These animals, which had to be euthanized on welfare grounds, had evidence of extensive systemic infection, with MU DNA detected in all organs tested. They also had multiple lesions present, which often extended deep into the underlying muscles and tendons and even exposed bones in two animals.

In cases where transmission occurs by mosquito bite, it is expected that animals would receive a lower inoculum dose than applied here^16^. As lower doses likely increase the incubation period and time to disease progression, this could also allow more time for MU to disseminate and potentially be shed in the feces. This may also explain why the only animal in the current study to show evidence of fecal shedding was the one that survived longest after development of clinical signs. However, the absence or near absence of AFB in internal organs may be temperature related. MU grows optimally between 30 and 32 °C and is often difficult to culture > 35 °C^34,35^. As the body temperature of the ringtail possums in this study was usually > 35 °C, this likely limited systemic bacterial growth. When Victorian MU isolates were grown at 37 °C, the bacterial population was reduced by 90% within 3–6 days^36^. We propose that the absence of MU DNA in the saliva in the current study, except at early time points, may reflect animals grooming the challenge site. Buccal swabs and/or salivary glands were positive in 9 of 27 naturally infected possums^12^. This may again reflect a lack of time for extensive dissemination of MU in our study animals or suggest that naturally infected animals acquire MU infection via an alternative route (e.g. ingestion). Rats that were orally challenged with high doses of MU subsequently expressed MU DNA both within and beyond their digestive systems, with samples of cervical, mesenteric and axillary lymph nodes, serum and spleen testing PCR positive^25^.

The ulcerated lesions noted in this study were histologically similar to those described in naturally infected humans, possums, and other animal species: primarily characterized by a deep necrotizing pyogranulomatous to granulomatous dermatitis and panniculitis, with abundant extracellular AFB concentrated in areas of tissue necrosis^12,13,18,37,38^. Under natural conditions lesions likely provide sites for opportunistic secondary infection by other pathogenic bacteria as observed in humans^39^. Where they extend into joints or bones, they may impact a possum’s mobility, behavior and risk of predation, contributing to both morbidity and mortality.

Also noted in this study was the regional spread of the inflammatory process from the inoculation site, systemic dissemination of the bacterium, as well as evidence of vascular thrombosis adjacent to the primary necrotic focus with presumably resultant ischemic damage to the suprajacent skin. Inflammation was seen to extend proximally from the point of inoculation via way of the subcutaneous adipose tissue, tendon sheaths, lymphatic vessels and, in one animal, blood vessels. Evidence of regional lymphatic spread with florid lymphangitis was evident in the proximal tail of four of the study animals and, in one of these, there was also evidence of vascular invasion with intraluminal AFB detected. Lymphatic and/or hematogenous pathways are considered the likely means for the systemic dissemination (hepatitis and/or pneumonia) noted in three possums. The impact of this systemic disease on ringtail possums is currently unknown. AFB were detected beyond the primary inoculation in only one possum (#2), and this was the only possum in which there was evidence of necrosis in inflammatory foci beyond the primary lesions. The mechanics of local and systemic MU dissemination remain imperfectly understood, but necrosis of regional lymph nodes and contiguous cortical bones underlying or even distant to severe skin lesions is known to occur in experimentally infected animals and occasionally humans^33,38,40^. The 1954 study that subcutaneously inoculated crude material extracted from a human patient’s BU into a rat’s tail observed the progress of the resultant ulceration over 12 months, noting that the lesion extended proximally and distally from the inoculation site and ultimately resulted in complete separation of the tail^18^. It remains unclear as to the significance of the noted vascular thrombosis and regional ischemia evident in this infection model to those seen with natural disease. Certainly, the lesions seem to follow a very similar clinical progression both macro- and microscopically and, as such, it would seem justified to implicate this as part of the pathogenesis in BU as per previous authors^41,42^.

This study reports the development of a ringtail possum model of MU infection and confirms that clinical BU can be replicated in this natural host species under controlled conditions. This model could be used to better understand the natural history of the disease, its pathogenesis, immunology and systemic spread of MU in this species, and potentially provide insights into the disease in humans. It would also allow potential transmission routes and mechanisms to be assessed, which could shed light on the epidemiology and ecology of this important yet poorly understood disease. Finally, as ringtail possums contribute to the circulation of MU in Victorian endemic areas, and their presence and infection status significantly increase the risk of BU disease acquisition in humans^11,43^, this model provides an opportunity to develop novel intervention strategies such as oral-bait vaccination of possums against MU^19^. Although such a strategy would ultimately be intended to benefit human health, prevention of MU infection in possums would represent a One Health approach, with added welfare benefits for possums that suffer considerable morbidity and mortality from this disease under natural conditions^12,13^.

## Methods

### Ethics statement

The animal ethics committee (AEC) of CSIRO’s Australian Centre for Disease Preparedness approved all animal work under approval number AEC: ACDP22012, in accordance with the National Health and Medical Research Council Australian code for the care and use of animals for scientific purposes 8th edition (2013)^44^. As ringtail possums are a protected species in Victoria, Australia, this work was conducted under Department of Environment Land, Water and Planning research permit number DELWP: 10,010,501. This study is reported in accordance with ARRIVE guidelines.

### Challenge material preparation and enumeration

*M. ulcerans *strain JKD8049, an Australian human clinical isolate collected in the Bellarine Peninsula in 2004, was used as the challenge material. This isolate has been extensively characterized for the purposes of standardizing challenge dosing, as described elsewhere^45^. In brief, the organism was cultured at 30^o^C in Sauton’s media (with animal-free supplement) in an orbital shaker, mechanically de-clumped by filtration, and stored in 20% glycerol cryopreservative. Colony forming units (CFUs) were determined by spotting 20 μm replicates of the thawed and briefly vortexed sample onto 7H10 Middlebrook agar supplemented with oleic acid, albumin, dextrose and catalase (OADC) in serial dilution. Agar plates were cultured at 30^o^C in atmospheric conditions for 18 weeks and CFUs were manually counted every two weeks until no new CFUs were apparent. Due to *M. ulcerans* culture being a prolonged process, culture was not attempted from samples collected from the animals challenged with *M. ulcerans* in this study.

### Trapping of wild possums

Animals were sourced from private properties in the Greater Geelong region. Prior to trapping, ringtail possum fecal samples were collected from potential properties and screened by detection of the MU-specific insertion sequence IS*2404 *PCR for MU DNA^46^. Trapping was only conducted at IS*2404* PCR negative properties. Ringtail possums were trapped using cage-style possum traps (61 × 18.5 × 19 cm) set overnight and baited with peanut butter and oat balls as well as apple. Upon capture the next morning, animals were health-checked by a veterinary professional and if deemed suitable for the challenge experiment, were transferred to individual wooden nest boxes (52 × 25.5 × 23 cm), provided with additional food (apple) and placed in a quiet, secure and sheltered location while awaiting testing outcomes. Fresh, voided fecal samples produced by the animals overnight were collected and screened by IS*2404* PCR for MU DNA. Only adult or subadult animals negative for MU DNA, over 350 g in weight, with no dependent young, no significant injuries and determined as visibly healthy by a veterinary professional were selected for use in the challenge study. Healthy juvenile animals or adults with dependent young were released at the site of capture at dusk of the same day, as were animals found positive for MU DNA but otherwise visibly healthy. Suitable animals (as described above) were transferred later the same day to the LAF at the Australian Centre for Disease Preparedness. This was repeated until a total of six suitable animals had been captured.

### Housing and acclimatization of possums

On arrival at the LAF, each nest box containing an individual animal was securely hung in a separate ‘aviary-style’ enclosure (1.5 m wide x 0.75 m deep x 1.9 m high) and the door to the nest box was opened. Within the enclosure animals were provisioned with water, various food items, several branches for climbing, a hanging basket as an alternative nesting location, and fresh foliage. All six animals were housed in separate enclosures in the same room, which was maintained at PC2 throughout the experiment. Animals were allowed to acclimatize for up to four weeks prior to experimental challenge. During acclimatization, animals were not physically handled by staff (except on health grounds) but were monitored overnight for activity and behavioral changes using infra-red motion capture cameras. Cages were swept out and water and treat items replenished daily, whilst fresh foliage was replaced every two to three days. This replenishment regime continued throughout the study.

### Experimental *M. ulcerans* challenge study

As this was an observational study assessing whether ringtail possums could be experimentally infected with MU, no negative control animals were included to minimize animal use. A sample size of six animals was chosen to minimize animal use and so that consistent infection could be demonstrated in a majority of animals (i.e. >75%). This was based on the assumptions that (a) one animal might not acclimatize to captive conditions, leaving five animals, and (b) that challenge failure may occur in one of these remaining five animals, providing a success rate of 80%.

On day 0 of the MU challenge, study animals were anaesthetized (inhalant isoflurane in oxygen, delivered via face mask) and health checked with the latter comprising a physical examination, assessment of weight, a subjective judgement of body condition^47^ and recording of rectal temperature. Baseline oral swabs, voided feces and blood samples were collected, and a temperature-sensitive microchip was implanted subcutaneously in the inter-scapula region for more accurate measurement of body temperature. The challenge site (dorsal surface of the mid-tail region) was then shaved and disinfected (70% ethanol) and animals were challenged intra-dermally with 0.2 ml of inoculum containing 506 CFU (95% confidence interval 463–551 CFU) of the Australian JKD8049 strain of MU. Animals were monitored for seven nights post-challenge using infra-red motion-sensor video cameras to check for any behavioral changes. These cameras were also used to monitor animals suspected to have health issues, reduced activity, or reduced food intake throughout the study.

Post-challenge, fresh voided fecal samples were collected daily from the floor of enclosures and tested by IS*2404* PCR. Prior to the onset of clinical signs, animals were anaesthetized and health checked (as described above with the addition of microchip temperatures being recorded) once weekly. Oral swabs and blood samples were collected at this time and tested by IS*2404* PCR, and the site of challenge was assessed for evidence of swelling, hyperemia, hair loss, surface erosion, or ulceration. Once changes were detected at the site of inoculation or adjacent to it, health checks and sample collection were increased to twice weekly, and swabs of the challenge site were also collected. Clinical signs were graded as mild, moderate or severe and are defined in Table 4. Animals were humanely euthanized by intravenous or intracardiac overdose with 325 mg/ml pentobarbitone at a dose of 150 mg/kg while deeply anaesthetized with Tiletamine/zolazepam (5-10 mg/kg) once one or more moderate (or severe) clinical signs were seen. This method of euthanasia is best practice for euthanasia of possums (as developed by experienced Australian wildlife veterinarians) and complies with the Australian code for the care and use of animals for scientific purposes^44^. This humane endpoint was chosen to allow confirmation of the causative agent of any lesions present and some investigation of disease pathology, without adversely impacting animal welfare in a severe manner. Ulcerative lesions in humans and mice are typically painless because MU bacteria produce mycolactone, a toxin that damages tissues and causes nerve degeneration, with the latter preventing transmission of pain signals^48^. Therefore, setting ulceration as a moderate clinical sign means that animals with small, simple ulcers are unlikely to have experienced any significant pain prior to euthanasia. Necropsies were conducted to look for evidence of gross pathology. The tail was removed from each animal at the sacrum (i.e. root of the tail), the tissue decalcified, and a full thickness transverse section was taken. During necropsy, samples from a range of tissues were collected for molecular testing and histopathology. Where histology revealed evidence of pathology, but molecular results were negative, the formalin-fixed paraffin-embedded (FFPE) samples used for histology were also subjected to molecular analysis. The variation in time to the development of swelling at the site of challenge, ulceration and euthanasia between study animals was assessed using descriptive statistics (i.e. mean, standard deviation).

**Table 4:** Grading of clinical signs in ringtail possums experimentally challenged with *M. ulcerans*.

Severity	Clinical sign
Mild	Subcutaneous swelling (nodule) +/- localised hair loss, superficial erosion at challenge site (‘pre-ulcerative’ lesion)
	Fecal or oral swab material positive for *M. ulcerans* DNA
Moderate	Swelling enlarges and/or becomes ulcerated
	Additional ulcers developing distant from original lesion (which may be at different stages of development)
Severe	Ulcer enlarges and/or extends more deeply with exposure of the underlying muscle seen
	Fluid accumulation around the margin of an ulcer
	Poor body condition score (i.e. a score of 2 or less)
	Reduced activity for > 2 nights

### Molecular testing

Molecular testing of all samples, except FFPE samples, was conducted as described previously (Blasdell et al., 2022). In brief, all sample types were collected into 2 ml tubes containing DNA/RNA Shield (Zymo, Irvine, CA) and a mixture of 2.3 mm and 0.5 mm zirconia/silica beads (Bio Spec Products, Bartlesville, OK). All sample types were homogenized, clarified and total nucleic acid extracted using the Quick DNA/RNA MagBead Pathogen kit (Zymo), followed by testing with the IS*2404 *real-time PCR assay, the standard assay for molecular detection of MU^46^. Samples producing a C_T_ value < 40 (threshold 0.02) were considered positive. FFPE samples were extracted using the Quick DNA/RNA MagBead kit (Zymo) as per the manufacturer’s protocol for FFPE samples, followed by testing with the IS*2404* real-time PCR assay, as above.

### Histology

After fixation of the tissues in 10% neutral buffered formalin, tissues were trimmed, processed routinely (ASP 300 S tissue processor, Leica Biosystems, Australia), and subsequently embedded into paraffin blocks. 4 μm thick tissue sections were then prepared and stained with haematoxylin-eosin and the Wade-Fite modification of the Ziehl-Neelsen staining method^49^.

### Sodium dodecyl-sulphate polyacrylamide gel electrophoresis of *M. ulcerans*

To prepare whole cell lysate, 4 mg of bacterial colony (grown on solid Sauton’s media with vegetable supplement) was collected into ZR BashingBead Lysis Tubes (0.1 & 0.5 mm; Zymo) containing 400 µL lysis buffer (PBS supplemented with 5% SDS and 1x Protease Inhibitor Cocktail (Abcam, Cambridge, UK)). Bacterial cells were homogenized using a mechanical bead beater device (Tissue Lyser; Qiagen, Hilden, Germany) twice at 6,800 rpm for 30 s. Tubes were centrifuged at 10,000 g for ten minutes and cleared lysate was removed into new tubes, with 1:10 and 1:100 dilutions prepared using PBS.

For sodium dodecyl-sulphate polyacrylamide gel electrophoresis (SDS-PAGE), undiluted MU whole cell lysate (15 µL) was added to 4x Sample Buffer (5 µL; Bolt LDS Sample Buffer; Thermo Fisher Scientific, Waltham, MA) and heated at 95 °C for five minutes. Whole cell lysate samples (20 µL) and a protein standards ladder (5 µL; Bio-Rad, Hercules, CA) were run on NuPAGE 4–12% Bis Tris gels (Thermo Fisher Scientific) at 200 V for 35 min. Gels were either stained with Coomassie Blue (Thermo Fisher Scientific) and visualized using ChemiDoc gel visualization system (Bio-Rad) or used for Western Blot analysis.

### Detecting seroconversion of possums to *M. ulcerans* using Western Blot and Dot Blot analysis

To investigate seroconversion to MU, selected samples were subjected to either Western Blot (WB) or Dot Blot analysis. Due to the lack of commercially available ringtail possum-specific secondary antibodies, *Staphylococcus aureus* Protein A-HRP conjugate was used as a secondary antibody substitute in these experiments. Possum #2 was selected to test for seroconversion via WB. Separated MU whole cell lysate proteins were transferred from SDS-PAGE gel to Invitrogen iBlot 0.2 μm Nitrocellulose Transfer Packs (Thermo Fisher Scientific) using the iBlot 2 Transfer System (Thermo Fisher Scientific) and the following program: 20 V for one minute, 23 V for four minutes, 25 V for three minutes. Following transfer, membranes were washed (washing Buffer; PBS supplemented with 0.1% Tween 20) for five minutes at room temperature (RT) and then blocked (blocking buffer; PBS supplemented with 5% skim milk powder and 0.1% Tween 20) for one hour at RT. Membranes were washed twice before incubation with possum serum. Possum #2 pre-infection and necropsy serum samples were diluted to 1:30, 1:100 and 1:1000 in blocking buffer and incubated with membranes overnight at 4 °C. Membranes were washed twice before incubation with *S. aureus* Protein A-HRP conjugate (1:2000 diluted in blocking buffer; Abcam) for one hour at RT. Membranes were washed twice then incubated with Pierce ECL Western Blotting Substrate (Bio-Rad) for one minute and visualized using ChemiDoc blot visualization system (Bio-Rad).

Dot Blot analysis was executed using MU whole cell lysate prepared as described above, using samples from Possum #3 as the primary probe. This method probes the entire whole cell lysate (WCL) sample, instead of separated proteins as in the WB. Bacterial lysate (Neat, 1:10 and 1:100 dilutions; 2 µL) was pipetted in duplicate onto nitrocellulose membrane and allowed to dry for one hour at 4 °C. Membranes were blocked with 5% BSA in PBS-T (PBS supplemented with 0.05% Tween 20) for one hour at RT. Possum #3 pre-infection and necropsy sera samples were diluted with 0.1% BSA in PBS-T to 1:10 (necropsy serum only) and 1:100, and incubated with membranes overnight at 4 °C. The no sera control was incubated with BSA/PBS-T containing no possum sera. Following incubation, the membranes were washed three times with PBS-T before incubation with Protein A-HRP conjugate (Abcam) diluted to 1:2000 in BSA/PBS-T for one hour at RT. Membranes were washed three times and blots visualized with Pierce ECL Western Blotting Substrate (Bio-Rad) and ChemiDoc blot visualization system (Bio-Rad).

## Electronic supplementary material

Below is the link to the electronic supplementary material.

Supplementary Material 1

## Data Availability

All data supporting the findings of this study are available within the paper and its Supplemental Information.
