# Supplementary material for: Experimental infection of ringtail possums (Pseudocheirus peregrinus) with Mycobacterium ulcerans, the agent of Buruli ulcer
Source: Sci Rep. 2024 Oct 25;14:25352. doi: 10.1038/s41598-024-76857-1 (PMC11511880; doi:10.1038/s41598-024-76857-1)
Supplement: Supplementary file 1 — Supplementary Material 1 [file 41598_2024_76857_MOESM1_ESM.pdf]

Supplementary information for:

Experimental infection of ringtail possums (*Pseudocheirus peregrinus*) with *Mycobacterium ulcerans*, the agent of Buruli ulcer

Kim R. Blasdell[1*], Richard J. Ploeg[2], Emma C. Hobbs[3], Stephen Muhi[4], Sarah J. Riddell[2], Alexandra Cunneen[2], Michael L. Kelly[2], Kate Maynard[2], Tess R. Malcolm[1], Md. Tanjir Islam[1], Victoria Boyd[1], Timothy P. Stinear[4], Sacha J. Pidot[4], Eugene Athan[5], Daniel P. O'Brien[5]

1: Health and Biosecurity, Commonwealth Scientific and Industrial Research Organisation, Geelong, Australia

2: Australian Animal Health Laboratory, Commonwealth Scientific and Industrial Research Organisation, Geelong, Australia

3: Department of Veterinary Biosciences, University of Melbourne, Melbourne, Australia

4: Peter Doherty Institute for Infection and Immunity, University of Melbourne, Melbourne, Australia

5: Department of Infectious Diseases, Barwon Health, Geelong, Victoria, Australia

*Corresponding author: kim.blasdell@csiro.au

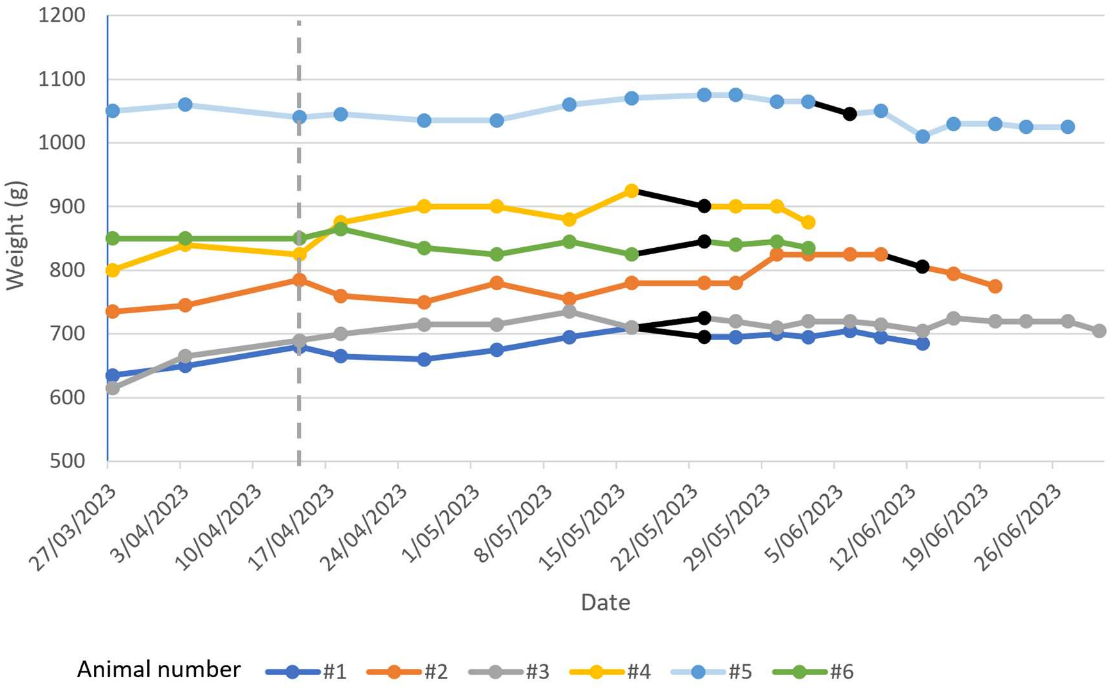

Supplemental Figure 1: Weight changes by animal, with challenge date denoted by a dashed grey vertical line and date of onset of lesion development shown in black.

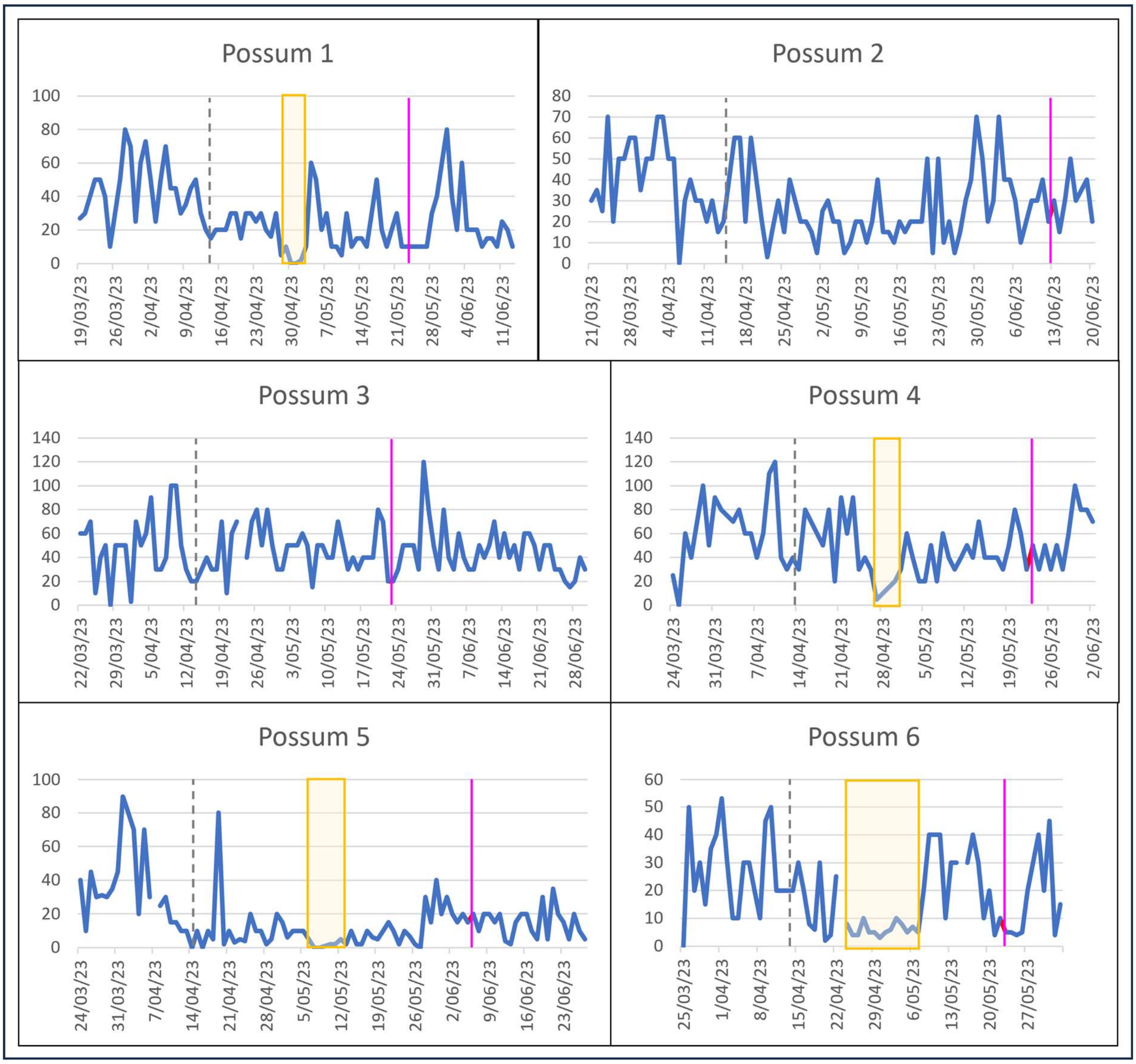

Supplemental Figure 2: Daily faecal production by animal with number of faecal pellets produced on the Y axis and date on the X axis. Challenge date is denoted by a dashed grey vertical line and date of onset of lesion development by a solid magenta vertical line. The periods of possible reduced faecal production are highlighted by a yellow box.

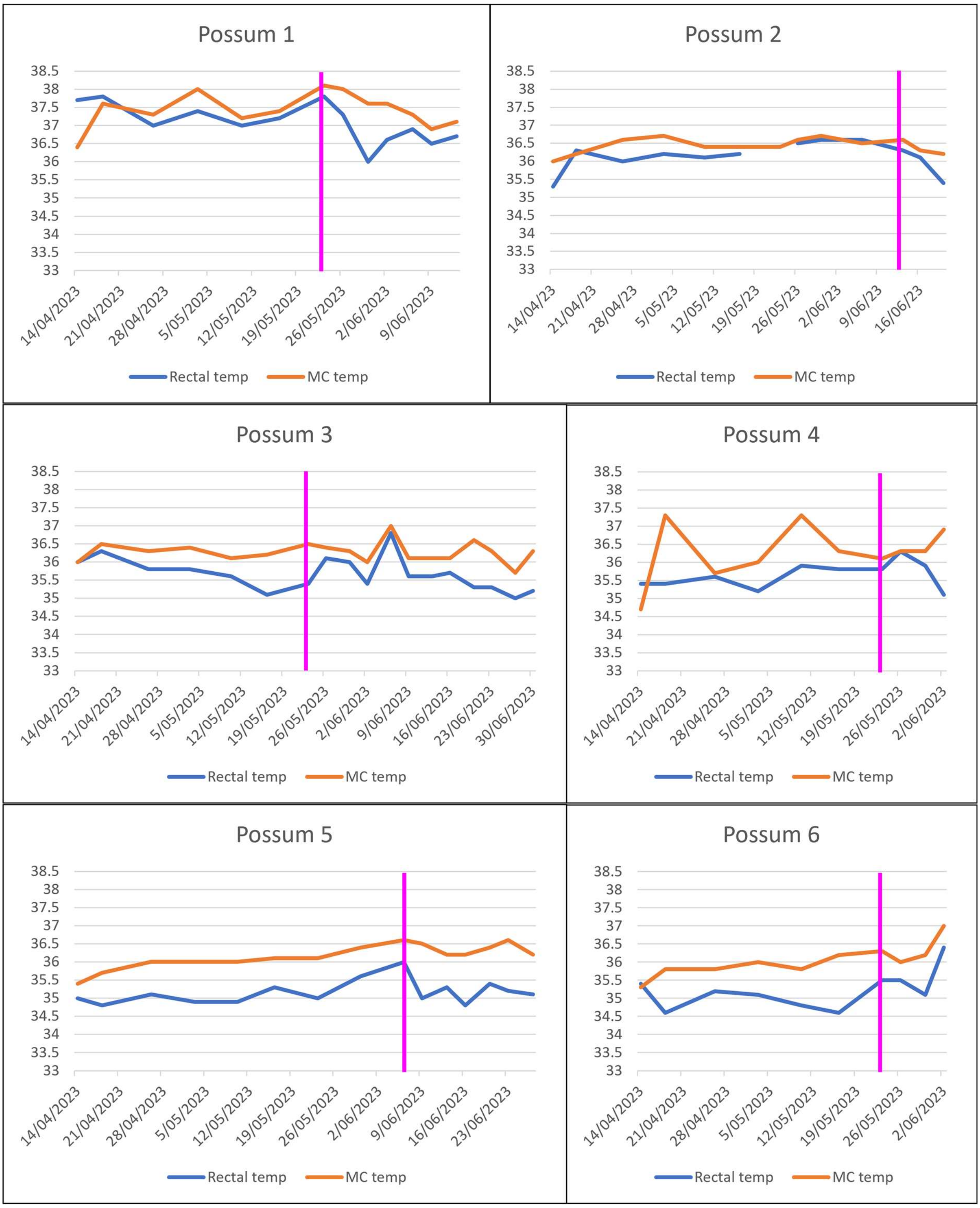

Supplemental Figure 3: Rectal temperatures (blue) and microchip (MC, orange) temperatures by animal from the date of challenge, with degrees Celsius on the Y axis and date on the X axis. The date of onset of lesion development is denoted by a solid magenta vertical line.

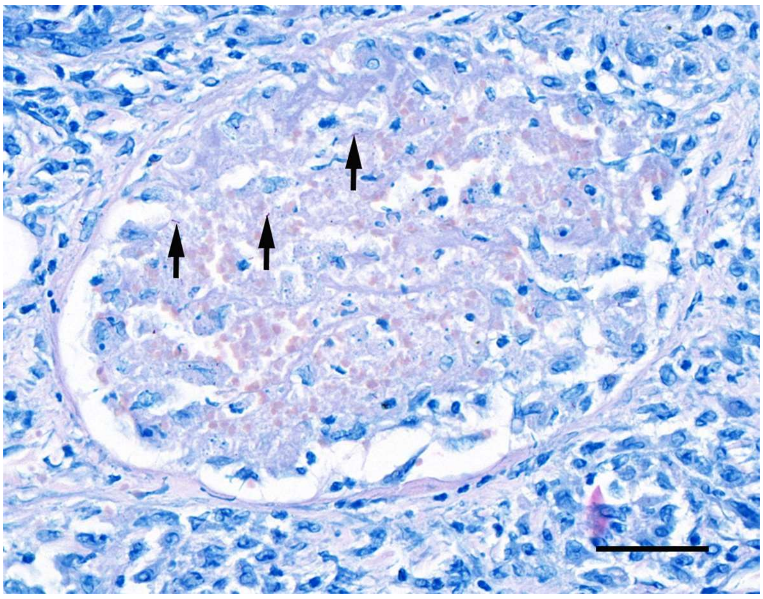

Supplemental Figure 4. Histology of a cross section of an inflamed blood vessel previously highlighted in Figure 4. Amidst the intra-luminal accumulation of necrotic debris and erythrocytes are scattered, extracellular, magenta-staining, individual acid-fast bacilli (arrows). Wade-Fite modification of Ziehl-Neelsen stain. Scale = 50µm.

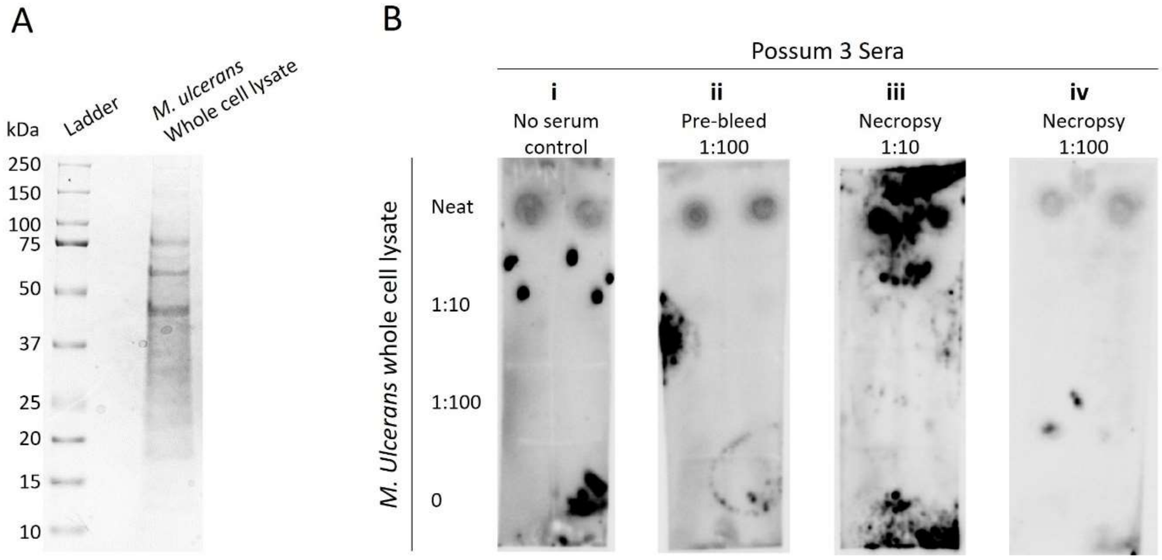

Supplemental Figure 5. (**A**) SDS-PAGE of M. ulcerans whole cell lysate. (**B**) Dot Blot to detect seroconversion of Possum 3 to MU. Bacterial whole cell lysate was used at neat, 1:10 and 1:100 dilutions, and no bacteria control, and probed with (i) no serum control, (ii) pre-infection bleed sera diluted 1:100, (iii) necropsy bleed sera diluted 1:10, and (iv) necropsy sera diluted 1:100. Blots were visualised using Pierce ECL Western Blot substrate and ChemiDoc visualisation system.
